# Development of a forward genetic screen to isolate oil mutants in the green microalga *Chlamydomonas reinhardtii*

**DOI:** 10.1186/1754-6834-6-178

**Published:** 2013-12-02

**Authors:** Caroline Cagnon, Boris Mirabella, Hoa Mai Nguyen, Audrey Beyly-Adriano, Séverine Bouvet, Stéphan Cuiné, Fred Beisson, Gilles Peltier, Yonghua Li-Beisson

**Affiliations:** 1 CEA Cadarache, Institute of Environmental Biology and Biotechnology, Saint-Paul-lez-Durance F-13108, France; 2 CNRS, UMR7265, Saint-Paul-lez-Durance F-13108, France; 3 Aix-Marseille Université, Saint-Paul-lez-Durance F-13108, France; 4 Present address: Institut des Sciences Moléculaires de Marseille, UMR 7313, Aix-Marseille Université, Marseille, France

**Keywords:** *Chlamydomonas* mutants, Flow cytometry, Genetic screen, Lipid remobilization, Microalgal oil, Nile red

## Abstract

**Background:**

Oils produced by microalgae are precursors to biodiesel. To achieve a profitable production of biodiesel from microalgae, identification of factors governing oil synthesis and turnover is desirable. The green microalga *Chlamydomonas reinhardtii* is amenable to genetic analyses and has recently emerged as a model to study oil metabolism. However, a detailed method to isolate various types of oil mutants that is adapted to *Chlamydomonas* has not been reported.

**Results:**

We describe here a forward genetic approach to isolate mutants altered in oil synthesis and turnover from *C. reinhardtii*. It consists of a three-step screening procedure: a primary screen by flow cytometry of Nile red stained transformants grown in 96-deep-well plates under three sequential conditions (presence of nitrogen, then absence of nitrogen, followed by oil remobilization); a confirmation step using Nile red stained biological triplicates; and a validation step consisting of the quantification by thin layer chromatography of oil content of selected strains. Thirty-one mutants were isolated by screening 1,800 transformants generated by random insertional mutagenesis (1.7%). Five showed increased oil accumulation under the nitrogen-replete condition and 13 had altered oil content under nitrogen-depletion. All mutants were affected in oil remobilization.

**Conclusion:**

This study demonstrates that various types of oil mutants can be isolated in *Chlamydomonas* based on the method set-up here, including mutants accumulating oil under optimal biomass growth. The strategy conceived and the protocol set-up should be applicable to other microalgal species such as *Nannochloropsis* and *Chlorella*, thus serving as a useful tool in *Chlamydomonas* oil research and algal biotechnology.

## Background

Declining fossil fuel reserves and increasing concern over greenhouse gas emissions have shifted research focus significantly toward the production of energies based on biomaterials. Energy production from microalgae has attracted the most attention due to the high biomass productivity of these organisms and minimal requirement for agricultural land use [1,2]. Many microalgae species have been found to synthesize large quantities as well as variety of fatty acids and lipids [1,3,4]. However, sustainable industrial production of bioenergy from microalgae has yet to be realized due to limitations at the biological level (robust strains, high oil yields, and so on) as well as at the technological side (cost of growing, cell harvesting, lipid extractions, etc.) [5]. On the biological side, one of the most challenging issues is to improve lipid productivity. Indeed, most microalgae make a large amount of oil (that is, triacylglycerols, TAGs) when subjected to stress conditions such as nitrogen depletion, which limits biomass productivity and thus overall lipid productivity [1]. Oil content engineering in microalgae has lagged behind that of plants [6], partly due to lack of knowledge on specific targets (enzymes, structural proteins, regulatory factors) of the lipid metabolic pathways. Thus, identifying genetic factors that regulate oil accumulation in algae will constitute a key step toward understanding and manipulating oil content in algal cells.

Genetic mutant screening by direct metabolite analyses has been used successfully to study numerous biochemical pathways in organisms ranging from bacteria to yeast and to higher plants [7]. Forward genetic screen is unbiased by preconceived concepts. It thus provides promises for assigning functions for genes where a biochemical activity of the gene product cannot be predicted based on sequence homologies to other known proteins. This is especially true for lipases, which play a key role in oil turnover in plant cells as reviewed by Troncoso-Ponce *et al*. [8]. Since *Chlamydomonas* diverged from the land plant over one billion years ago [9], a forward genetic approach in a microalga will likely provide opportunities to discover unique algal pathways [10].

Although microalgae have a high level of biodiversity, only a few species can be subjected to genetic manipulation [6]. The alga with the best developed genetic toolbox is the unicellular green microalga *C. reinhardtii*[11]. It is a well-established model for the study of various cellular processes especially photosynthesis, flagella, starch metabolism and photobiological production of hydrogen [11]. Like many other algal species, *C. reinhardtii* can accumulate significant amount of oil when subjected to unfavorable environmental conditions [12-16]. *C. reinhardtii* has proven an excellent model to study basic questions relating to the improvement of microalgae for biodiesel production, and substantial literature related to lipid metabolism has just started to emerge in this model alga [17,18]. It is unicellular and stays as haploid during most of its life cycle [11], thus is particular useful in the context of a forward genetic approach because the mutant phenotype can be observed during the first generation and does not need to reach a diploid homozygous stage.

Genetic mutant screening by direct lipid analyses has also been applied in *C. reinhardtii*, and led to the isolation of *sqd1*[19], *pgd1*[20] and the *crfad7* mutants [21], and some other mutants currently under characterization [22]. However, isolation of mutants with robust phenotypes in oil accumulation or remobilization is not an easy task in *Chlamydomonas* since the cellular oil content is highly variable, not only between genotypes but also depending on chemical or physical environmental stimuli, growth phases or aging of the culture [1,14].

In this study, we report the screening of an insertional mutant library of *C. reinhardtii* based on direct detection of oil content. The culture conditions adapted for high-throughput approaches, the three-step procedure used to improve screening efficiencies in *C. reinhardtii*, and the results of the screen are described. This study serves as a first detailed guide on screening of oil mutants in *C. reinhardtii*.

## Results and discussion

### Concepts and types of mutants searched for

To identify factors that are critical to oil accumulation and turnover, thus providing molecular tools for genetic engineering studies, we set up a genetic screen to isolate mutants affected in oil content in the model alga *C. reinhardtii*. Our overall screening strategy was based on the dynamic process of oil accumulation and remobilization under different nitrogen statuses. The same culture were subjected to three time-resolved nutrient statuses: first, optimal growth conditions (Tris-acetate-phosphate (TAP) + nitrogen (N)/light); second, nitrogen starvation condition (TAP-N/light); then third, the remobilization condition (minimal medium (MM) + N-acetate/dark). A fraction of cells was taken at each condition, which allowed isolation of three types of mutants, as follows.

#### Type I mutants (Screen I)

Under optimal growth conditions, wild-type *Chlamydomonas* strains accumulate very low amounts of oil (<1 μg per 10^6^ cells [14]; and Additional file 1). Type I mutants refer to those that show an increased oil amount under optimal growth conditions. Isolation of this type of mutant will allow us to decouple oil accumulation from the requirement for stress, providing strains that produce oil and biomass simultaneously, thus increasing the overall lipid productivity.

#### Type II mutants (Screen II)

When cells are subjected to stresses such as nitrogen starvation, oil content can be increased more than 10 fold (up to 10 μg per 10^6^ cells) [12-14]. This is consistent with the extensive metabolic shift toward carbon reserve formation under nitrogen deplete conditions [23]. Type II mutants refer to those that are isolated under nitrogen-depleted conditions (TAP-N). Although the oil accumulation process is well-characterized in *Chlamydomonas* in response to nitrogen starvation, little is known about the molecular players and its regulations. For example, only a couple of proteins (a diacylglycerol acyltransferase, a phospholipid:diacylglycerol acyltransferase, and one nitrogen regulator) have been experimentally demonstrated to contribute to oil accumulation [24,25], and no transcription factor has yet been identified. Isolation of mutants with altered oil content under nitrogen depletion should yield novel insights into the molecular mechanisms linking oil accumulation and carbon partitioning with response to stresses.

#### Type III mutants (Screen III)

In *Chlamydomonas* it has been demonstrated that the oil accumulated under nitrogen depletion can be degraded within hours of adding nitrogen back to the starved cells [14], which is in accordance with the presence of putative lipolytic enzymes associated with oil bodies [15,26]. Oil remobilization is a natural phenomenon observed in numerous microalgal strains upon re-establishment of optimal growth. Intracellular TAG amounts also fluctuate during the diurnal cycle because TAGs produced during the day provide a carbon and energy source for the night [27]. This could be a major factor contributing to yield loss because industrial production of microalgae uses sunlight as an energy source and is therefore subjected to the influence of day and night cycles. Thus, understanding the genetic basis of oil turnover, and further developing molecular tools to control this process, will be highly beneficial. Lipid turnover is likely a constitutive process in algal cells similar to that occurring in plant cells [8]. Type III mutants refer to those that are altered in oil remobilization after adding nitrogen back to the culture medium under dark conditions (MM/dark). Blocking oil turnover processes might help increase the level of oil accumulated, as was observed in *Arabidopsis* leaves where the oil content was increased 10-fold by knocking out a lipase gene [28].

### Generation of a tagged insertional mutant library

#### Parental strain

Although the entire laboratory strains of *C. reinhardtii* originate from a single strain [11], many spontaneous mutations have occurred since its isolation 60 years ago in Massachusetts. It has been recently estimated, based on whole genome resequencing, that there are >24,000 changes (including single nucleotide variations and insertions/deletions) between two common laboratory strains [29]. This high rate of mutation is largely due to the haploid genome and could potentially explain the variation in oil content among the so-called wild-type strains [14]. For any forward genetic screens, the suitability and quality of the parental strain is thus very important. In an oil content screening project, the parental strain needs to meet the following criteria: relatively high oil content, high rate of transformation, sensitive to antibiotics, and the possibility of being genetically crossed if needed. After comparing several strains, *dw15.1* was found to conform to all the criteria. In addition, this strain is defective in cell wall, which facilitates the transformation procedure (that is, there is no need to remove the cell wall by the lengthy autolysin treatment). The strain *dw15.1* was thus used as a host strain to generate the mutant library.

#### Choice of the tagging method

Mutant collections can be generated via various means including UV, chemical mutagenesis, or by insertion of a DNA fragment conferring resistance to antibiotics or overcoming a need for certain vitamins or amino acids. Because one of the time-consuming steps associated with forward genetic screens is the identification of the mutated gene, we have chosen as a first approach tagged insertional mutagenesis. This method uses transposons, antibiotic selection markers or transfer DNAs. An identifiable ‘tag’ is inserted into the host genome, which then allows identification of the position of insertion in the genome via PCR-based techniques. Various antibiotics have been used as markers for selection of transformants [30]. Among these, the *AphVIII* gene from *Streptomyces rimosus* coding for an aminoglycoside 3’-phosphotransferase, which confers resistance to the antibiotic paromomycin [31], was used as a selection marker for this study because the drug-resistant phenotype is stable [32]. The *AphVIII* cassette was delivered into the host genome via glass-bead-mediated transformation as reported in [33]. Using this method, on average >300 transformants were obtained per microgram of DNA.

### Design of the screening strategy

#### Estimation of oil amount: correlation between Nile red fluorescence and the amount of TAGs measured by thin layer chromatography

The conventional method of TAG quantification generally involves time-consuming steps including lipid extraction, separation, concentration and analysis, and is thus not suitable for a high-throughput screen. Here, we have taken advantage of the fact that cellular oil content can be estimated qualitatively by staining live cells with the lipophilic fluorescence dye, Nile red, a method widely used to monitor oil accumulation in different organisms [12,14,15,28,34,35]. Nile red staining combined with flow cytometry allows simultaneous screening of hundreds of independent transformants. Since Nile red fluorescence is only an indicator of oil amount and does not refer to the absolute oil content itself, we first evaluated the correlation of cellular oil contents determined by lipid extraction and thin layer chromatography (TLC; which gave absolute TAG quantity per cell) with that estimated by Nile red staining and flow cytometry (which indicates the level of Nile red fluorescence denoted as an arbitrary unit (AU)). Although a few previous studies have established a positive correlation between Nile red fluorescence and cellular oil content, these were mostly based on using oil standards or was only done on a number of limited pre-chosen algal strains [36].

**Figure 1:**
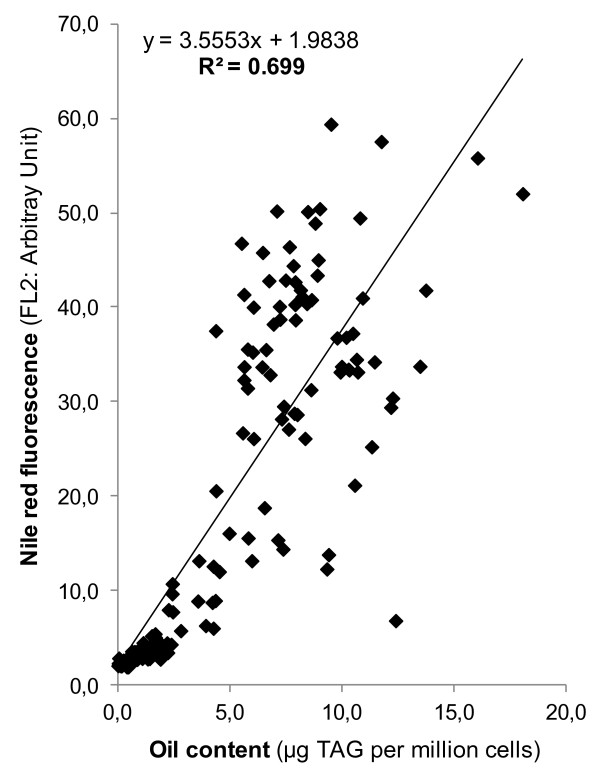
**The positive correlation of Nile red fluorescence and oil content determined by thin layer chromatography.** Randomly chosen insertional mutants (>100) were subjected to both analyses, and the datasets were compared via regression analysis in Excel. Each data point represents a mean of three biological replicates. FL2, fluorescent light channel 2.

In this study, to enlarge the coverage and better reflect the *in situ* screening situation, we randomly chose >100 transformants, and analyzed them by both Nile red/flow cytometry and TLC (Figure 1). Regression analysis gave an R square of 0.7 and a slope of the regression curve that was significantly different from 0 (*P* around 4e^-40^). This statistical analysis thus indicated the existence of a positive correlation between the levels of Nile red fluorescence and the actual oil content measured among randomly chosen clones. Furthermore, setting the cut-off value of fluorescent light channel 2 at 5 and the oil content at 3 μg TAG per million cells resulted in a rate of false positive of 8%, and a rate of false negative of 1%. Some occasional outliers between the two analyses could be due to mutations affecting various other parameters such as cell permeability to the dye, or intracellular structural modifications. Indeed, Nile red fluoresces in a lipid-rich hydrophobic environment, which does not necessarily result from an increase in TAG content but could also be caused, for example, by an increase in membrane curvature that potentially creates a micro-hydrophobic domain [34]. Furthermore, Nile red also renders hydrocarbons, wax esters, and also polyhydroxyalkanoic acids fluorescent [37,38].

#### Growth phases

Cellular oil content varies with growth phases [1]. For example, it has been shown that cells of the chlorophyte microalga *Parietochloris incisa* synthesize almost twice as many TAGs in the stationary phase than in the logarithmic phase [39]. To avoid any potential differences brought about by cell phases rather than by genetic mutations, we made serial multiple dilutions of the cultures (refreshment of cell cultures) to ensure that independent colonies would reach similar growth phases at the time of analyses. This was based on our observation that cell concentrations varied significantly in 96-deep-well plate cultures directly inoculated from a colony from an agar plate. Thus, routinely, after being inoculated from a single colony and grown for six days, cultures were diluted 30-fold to reach a concentration of 0.6 million cells per mL. After propagating for another two days, exponential phase cells were ready for analysis.

#### Position on the 96-deep-well plate

At the beginning of the screening, we hypothesized that depending on the position of the plate, cells could potentially be subjected to different light exposure thus bringing in variations in oil content. We tested this variability by inoculating all 96 samples with the same wild-type strain, and then measured Nile red fluorescence after four days of nitrogen starvation. The coefficient of variation of the level of Nile red fluorescence for all 96 wells was found to be 0.08, which indicated a reasonably small positional effect on the plate. This might be due to the presence of acetate in the culture medium, which probably minimized the dependence of culture growth on light (mixotrophic conditions).

#### Kinetics of oil accumulation and remobilization in 96-well plates

**Figure 2:**
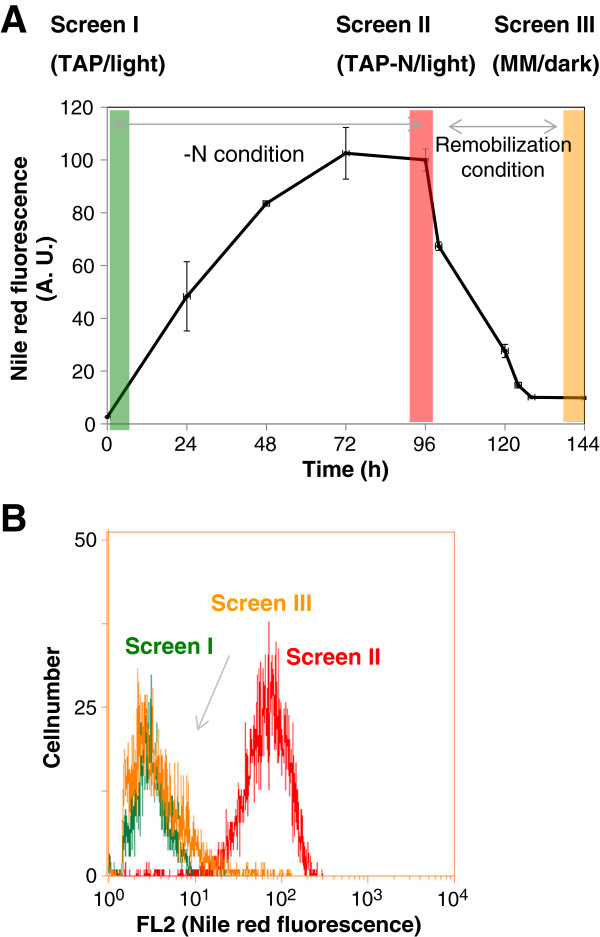
**Dynamics of oil accumulation and turnover in response to nitrogen status in microplate cultures. (A)** Changes in cellular oil content as estimated by the level of Nile red fluorescence. Shaded areas denote the three time points where the corresponding screening was performed. **(B)** Distribution of Nile red fluorescence at a population level for each time point sampled. AU, arbitrary unit; FL2, fluorescent light channel 2; MM, minimal media; N, nitrogen; TAP, Tris-acetate-phosphate.

The kinetics of oil accumulation and turnover so far reported in *Chlamydomonas* have been based largely on cells cultivated in shake flasks [14,40]. In this study, we therefore first analyzed the kinetics of oil accumulation and turnover when cultivated in 96-deep-well plates for the wild-type strain. This analysis also helped us to define the time point of sampling for the screening. Single colonies were first cultivated in a 96-deep-well plate in TAP medium until mid-log phase growth (for the nitrogen-replete condition), and then cells were transferred to TAP-N medium for the nitrogen-depleted condition. For seven days, a culture aliquot was taken each day, transferred to a new plate, stained with Nile red and further analyzed by flow cytometry. We observed that Nile red fluorescence levels continued to increase, albeit slightly, until the seventh day (data not shown). However, at this point, a large fraction of cells died and cultures turned yellowish. We found that 96 hours (that is, the fourth day) after nitrogen starvation is a good compromise between oil content and cell vigor. To induce oil remobilization, cells starved for nitrogen over four days were centrifuged and resuspended in MM and kept in the dark for up to two days. The kinetics of oil accumulation and remobilization in cells cultivated in 96-deep-well plates (Figure 2) were similar to that of shake flask cultures [14] and >80% of oil reserves was remobilized within 48 hours upon regain of nitrogen in the medium (Figure 2A). Shifts of Nile red fluorescence in response to nitrogen status (at the three sampling points, highlighted in Figure 2A) can be clearly observed at a cell population level (Figure 2B).

### Mutant screen

#### Procedure used

**Figure 3:**
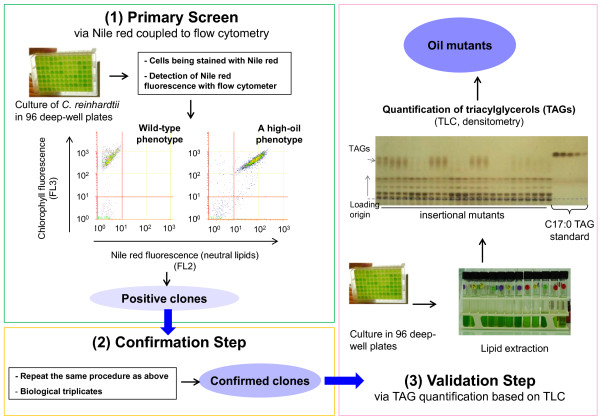
**Experimental outline of the three-step screen used to isolate oil mutants of *Chlamydomonas reinhardtii*.** FL, fluorescence light channel; TAG, triacylglycerol; TLC, thin layer chromatography.

We designed a screening procedure considering all the parameters described above. This approach consisted of three major steps (Figure 3): first, a primary qualitative screen based on cells grown in 96-deep-well plates and analyzed with Nile red and flow cytometry; second, a confirmation step involving biological triplicates grown in 96-deep-well plates and analyzed as in the first step; and third, final validation of the potential mutants via lipid extractions and oil content quantification by TLC. For the same batch of cell cultures, three time points were taken for analyses (see Figure 2A). It took less than 1 hour to read through one plate containing 96 samples. The mean fluorescence was calculated for the entire plate and all strains that fluoresced 50% more (or less) than the mean of the plate were retained for a second-round of flow cytometer analysis using triplicate biological samples. After passing two rounds of screening with Nile red and flow cytometry, selected clones were subjected to quantitative analysis of the oil content using lipid extraction and TLC as previously reported by our laboratory [14].

#### Summary of mutants isolated

**Figure 4:**
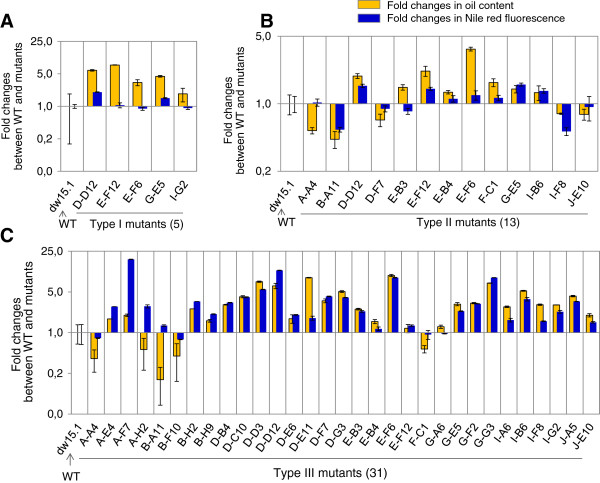
**Fold changes of oil content between wild-type and the mutants isolated.** Values are based on means of three replicates, and error bars represent the % of variation between biological triplicates. **(A)** Type I mutants isolated under TAP condition. **(B)** Type II mutants isolated under TAP-N condition. **(C)** Type III mutants isolated under MM condition. WT, wild-type.

After the first round of screening with Nile red and flow cytometry, a total of 76 viable mutants (4.2%) were isolated from the 1,800 transformants screened (Table 1). These 76 clones were retained for a second analysis with triplicate biological cultures for each strain. On the 76 clones, 41 were confirmed at this step. Oil quantification by TLC validated 31 clones for their oil content phenotypes. This demonstrated that a second-round analysis with Nile red and triple biological samples was essential to eliminate a substantial number of false positives (35 on 76, about 46%), thus constituting a critical step of the screening procedure. Among the 31 validated mutants, 5 contained higher oil content than wild-type under nitrogen-replete conditions (type I), 13 had altered oil content under nitrogen-depleted conditions (type II), and all 31 mutants showed changed capacity in oil remobilization (type III) (Figure 4). While in most cases, a positive correlation was present between the two methods, occasional discrepancies (for example, in the case of the mutant A-H2 and B-A11, see Figure 4C) could be due to false positive Nile red staining as explained previously in the text.

**Table 1:** A summary of mutants isolated at each step of the screen

**Types of mutants**	**Primary screen (Nile red/flow cytometry)**	**Confirmation step (Nile red/flow cytometry)**	**Validation step (Lipid extraction/TLC quantification)**
Type I	8 (0.4%)	8 (0.4%)	5 (0.3%)
Type II	37 (2.1%)	16 (0.9%)	13 (0.7%)
Type III	45 (2.5%)	39 (2.2%)	31 (1.9%)
Total number of mutants; overall rate	76 (4.2%)	41 (2.2%)	**31 (1.7%)**
Rate of confirmation between the sequential step of screen	-	54%	73%

The data x (y%) refers to the number of mutants that are grouped under each type (x), and the rate of their isolation based on a total of 1,800 transformants examined (y%). TLC, thin layer chromatography.

Two mutants (E-F12, D-D12) accumulated more than five times more oil than wild-type under nutrient sufficient conditions: oil content increased from 0.2 to 1.46 μg per million cells in E-F12 and to 1.12 μg per million cells in D-D12 (Figure 4 and Additional file 1). Although this level is still far below that under nitrogen-depleted conditions, it is nonetheless significant, demonstrating that genetically it is possible to increase oil accumulation under non-stress conditions. These mutants also grew normally as compared to wild-type. Within type II, eight mutants showed increased oil accumulation whereas five accumulated less oil per cell than their corresponding wild-type background. Between these strains, cellular oil content ranged from approximately 3 to 30 μg per million cells (Additional file 1). The mutant E-F6 accumulated 30.5 ±5.0 μg TAG per million cells (mean ± SD, *n* = 3) which is higher than all known high-oil accumulators so far reported for *Chlamydomonas*[14]. Further study of this mutant should yield important information on oil metabolism and its regulation. The wide variation in oil content between the mutants isolated indicates a high plasticity of *Chlamydomonas* cells to accumulate and accommodate oils, and this capacity can indeed be manipulated by genetic means.

Two days after switching to nitrogen sufficient conditions (from TAP-N/light to MM/dark), the wild-type *dw15.1* was able to reduce its cellular oil content from 8.3 ±1.9 to 1.4 ±0.5 μg per million cells (mean ± SD, *n* = 10). Among the 31 mutants affected in oil remobilization, 25 showed reduced capacity and 5 increased capacities in utilizing oil reserves compared to the wild-type (Figure 4 and Additional file 1). After two days, the amount of oil remaining ranged from 5% to 80% of that accumulation under nitrogen-depleted conditions. Compared to reactions of oil synthesis, oil remobilization is even less known, with only one *Chlamydomonas* lipase so far having been functionally characterized [40]. Detailed studies of these oil remobilization mutants isolated here should provide genetic insights into the pathways and factors involved in lipid turnover in an algal cell.

**Figure 5:**
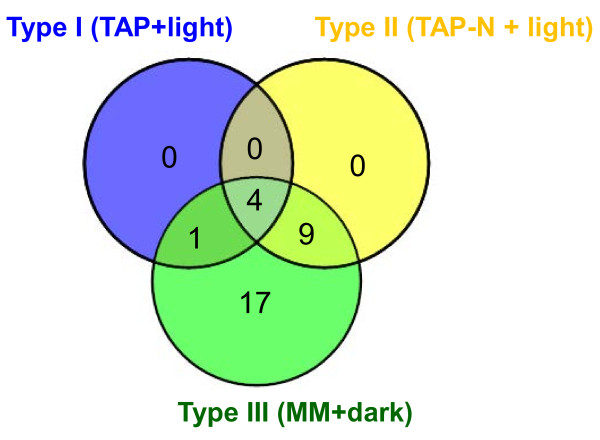
**Overlap between the three types of mutants obtained.** The Venn diagram was drawn using the online software VENNY designed by Oliveros [41]. MM, minimal media; N, nitrogen; TAP, Tris-acetate-phosphate.

Cross-comparison of mutant phenotypes revealed that all mutants of type I and type II also showed altered capacity to remobilize oil when switching to the oil remobilization condition (Figure 5). This observation gave support to the notion that intracellular oil accumulation results from equilibrium between synthesis and degradation processes, and that it is possible to disrupt this equilibrium via a genetic approach. This raises the question of why no mutants showing only type I or type II phenotypes were isolated during this study. One of the reasons could be that, in the current screen, we kept only clones that showed 50% more or less fluorescence than wild-type, which could have been a too stringent condition to obtain such mutants. For example, the mutants of *Chlamydomonas pdat1-1* and *pdat1-2* defective in the phospholipid:diacylglycerol acyltransferase, which is involved in TAG synthesis, accumulated only 25% less TAG compared to its parent strain [24]. Secondly, only approximately 1,800 mutants have been screened here, which is far from ‘saturating’ the genome. Thirdly, the presence of multiple parallel lipid biosynthesis pathways could cause redundancy, thus explaining a lack of type I only or type II only phenotypes. A fourth explanation could be that oil accumulation in the nitrogen-depleted condition has been shown to be essential to cell survival [20], thus mutants incapable of oil synthesis could be not viable.

The isolation of a large number of mutants affected in lipid remobilization (31) could reflect that disruption of not only factors governing lipid equilibrium but also other pathways (for example, lipid remodeling) could lead to such a phenotype. Lipid remodeling is a complex process, and likely controlled by complex regulatory networks. This could be related to the presence of a large number (130) of proteins containing a known ‘lipase’ motif, GXSXG, encoded in the genome of *Chlamydomonas*[23]. This is much greater than the number of lipid-related biosynthetic genes. Miller *et al*. [23] further observed that during the transition from nitrogen-replete to nitrogen-depleted conditions, of all lipid-related genes, those encoding putative lipases showed strongest differences in transcript abundance.

Identification of genetic bases of these mutations (via PCR-based techniques or whole genome resequencing) [29] and the further establishment of the genetic link between the observed oil phenotype and the affected genetic locus for each mutant isolated consist of genetic complementation or crosses, thus requiring substantial time and effort. Molecular genetics and physiological characterization of these mutants will be work on its own right, and will be reported in due course.

## Conclusion

In this study, we showed that flow cytometry analyses of Nile red stained cells is an efficient way to isolate mutants with affected oil content in *C. reinhardtii*. Applying this method, 31 mutants have been obtained and validated after screening of a first set of 1,800 transformants. This work shows that it is possible to isolate oil mutants in *Chlamydomonas* with a reasonable rate of success (1.7%). The overlapping phenotypes between the three screens point toward a likely central role of lipid turnover processes in the level of oil accumulated under nitrogen-replete or -deficient conditions.

The protocol reported should thus provide a basis for further functional genomic studies of oil metabolism in *Chlamydomonas* and serve as a reference for genetic screens of oil mutants in other microalgal models such as *Nannochloropsis* and *Chlorella*. The methodology set-up should also be adaptable to isolate mutants generated by other means, for example, UV, fast neutron or chemical mutagenesis. Point mutations or deletions could be identified by map-based cloning, which has already been established for *Chlamydomonas*[42].

## Materials and methods

### *C. reinhardtii* strains and culture conditions

The cell-wall-less *C. reinhardtii* strain *dw15.1* (*nit1-305 cw15; mt*^*+*^) was used in this study as a parental strain to generate the insertional mutant library. Unless otherwise stated, *dw15.1* and the mutants generated from it were maintained on agar plates containing TAP medium [11] supplemented with 2% agar (w/v), and in the case of mutant, 10 μg mL^-1^ paromomycin was added. The plates were kept in controlled growth chamber set at 25°C under continuous illumination (approximately 30 μmol photon m^-2^s^-1^). To prepare the medium without nitrogen (TAP-N), ammonium (NH_4_Cl) was omitted from the regular TAP medium described above.

Mutant libraries were maintained on TAP agar plates in the 96-well format. For liquid cultures, cells were cultivated in polypropylene 96-deep-well plates (polypropylene, 2 mL; V-shape, from Greiner Bio-One), which minimized cells sticking to the wall and allowed cultivation of cells in relative large volume (600 μL) with sufficient agitation, which was found to be important to avoid cell precipitation. The 96-deep-well plates were then sealed with an AeraSeal™ sterile film (Excel Scientific, Victorville, CA, USA) specific for cell cultivation, which keeps culture sterile while allowing gas exchange. These 96-deep-well plates were incubated in an Infors (Infors AG Rittergasse 27, CH-4103 Bottmingen/Basel, Switzerland) with the following parameters: growth temperature of 25°C, light density of 150 μmol photon m^-2^s^-1^ and agitation speed of 300 rpm. To minimize evaporation, it was observed that maintaining a high relative humidity (approximately 70%) in the cultivation chambers was essential.

For nitrogen starvation studies, the total culture was centrifuged at 600 *g* for 3 min at room temperature, washed once with TAP-N media, and then suspended into 580 μL TAP-N. To induce oil remobilization, cells were again centrifuged, and cell pellets were resuspended in MM (that is, an autophototrophic condition) [43], which has a similar composition to TAP except that acetic acid is replaced by hydrochloric acid (48.5 mM), thus MM lacks an organic carbon source which allows photoautotrophic growth. During the phase of oil remobilization, cells were kept in the dark.

### Generation of a random insertional mutant library for *C. reinhardtii*

An insertional mutant library was generated via transformation of the *AphVIII* gene conferring resistance to paromomycin into *dw15.1*. To increase efficiency and avoid multiple insertions or re-arrangement in the genome, not the whole plasmid, but only the DNA fragment containing antibiotic resistance was used for transformation [32]. The DNA cassette (about 1.8 kb) containing the *AphVIII* gene driven by the *RBCS2:HSP70A* hybrid promoter and terminated by the *RBCS2* terminator [31] was obtained by digesting the pSL18 vector with *Kpn*I and *Xho*I **(**New England Biolabs, Evry France). The digested plasmid was then migrated on a 1% agarose gel, and the fragment containing *AphVIII* gene purified using a Qiagen gel purification kit (Qiagen, Courtabeeuf, France).

Transformation was carried out using the glass bead method of Kindle [33]. Briefly, cells of *C. reinhardtii* were cultivated under standard conditions in TAP liquid media until reaching exponential phase (a cell concentration of 5 to 8 × 10^6^ cells mL^-1^). To ensure homogenous cell growth, cell cultures were refreshed a couple of times before being used for genetic transformation. For each transformation, 10^8^ cells were harvested by centrifugation (600 *g* for 2 min at room temperature), resuspended into 330 μL TAP and transferred to an Eppendorf tube containing around 300 mg sterile acid-treated glass beads (0.4 to 0.6 mm in diameter, Sigma-Aldrich, Saint-Quentin-Fallavier, France). To the cell mix, approximately 1 μg DNA was added, and vortexed for 16 s. The cell suspension was transferred gently with a 1 mL tip (cut at the end) avoiding taking any glass beads, then being spread on to a TAP agar plate containing 10 μg mL^-1^ paromomycin. The agar plates containing the transformants were kept under the laminar flow hood for another half an hour to evaporate excess medium. The plates were then sealed with a Parafilm and kept under dim light (*ca.* 10 μmol photon m^-2^s^-1^) in growth chambers for the first 24 h then in standard light conditions (*ca.* 60 μmol photon m^-2^s^-1^) afterwards. Antibiotic resistant colonies were seen after 7 to 10 days cultivation. All transformants were then picked up with a sterile wooden tooth pick and arranged in a fresh agar plate in 96-well format. This library was ready for screening. Like the agrobacterium-mediated Transfer-DNA insertion into plant genomes, the DNA cassette was inserted into the nuclear genome of *C. reinhardtii* in a random manner.

### Qualitative estimate of neutral lipids based on Nile red fluorescence and flow cytometry

Neutral lipids stained with Nile red (Sigma N3013) show a particular emission peak at 580 nm when excited at 488 nm by the laser [34]. Fluorescent signals can be detected under microscope, through a plate reader, or based on Flow Cytometer. Flow cytometry is a system based on the principle of light scattering, light excitation and emission of fluorochrome molecules to generate specific multi-parameter data from particles and cells simultaneously. In this study we used the Flow Cytometer (Cell Lab Quanta™SC, Beckman Coulter) to detect the fluorescence signal of Nile red and at the same time, multiple parameters (cell size, cell concentration, chlorophyll fluorescence, population distribution) were collected. Another major advantage of flow cytometry is that it can do multi-parametric analyses up to thousands of particles per second.

Briefly, cells grown to desired stage were transferred first from a 96-deep-well plate (where they were cultivated) to a microtitre plate (300 μL; V-shape, Nunc (Greiner Bio-One SAS)) and diluted with iso-Diluent (Beckman Coulter, Paris, France) accordingly to reach a final concentration of 0.5 to 2.5 × 10^6^ cells mL^-1^ in a total volume of 200 μL. Nile red was added to reach a final concentration of 4 μg mL^-1^ from a stock solution of 1 mg mL^-1^ dissolved in methanol. The reaction mixture was then incubated at 40°C under shaking for 10 min in the dark before analyses. Nile red is light sensitive; therefore all the preparation steps related to handling of the fluorescence dye were carried out under dim light.

After being stained, cells were excited at 488 nm by the laser. Emission signals were collected at different fluorescence light (FL) channels. Cell concentration and cell size were measured at the same time by impedance analysis. In our set-up, the FL2, collecting signals from a 575 nm bypass filter, was used to record the emission of Nile red once bound to neutral lipids; FL3, collecting signals from 670 nm bypass filter, recorded chlorophyll fluorescence. Iso-diluent (Beckman Coulter) was used throughout as a sheath fluid for the flow cytometry. The standard setting for the screening method in the flow cytometer was: 22 mW laser; flow rate 45 μL min^-1^. Analysis finished when the total number of analyzed cells reached 7,000, analytic volume reached 100 μL, or analytic time reached 12 s. Data were analyzed by Quanta Analysis software (Beckman Coulter software). Applying this method, one plate of 96 samples could be analyzed in under one hour.

### Quantification of oil content based on thin layer chromatography

To quantify oil content, total cellular lipids were extracted, and oil content determined after separation on TLC plates as described before [14].

### Statistics

Correlation analysis between the level of Nile red fluorescence and TAG quantification by TLC was calculated using the correlation regression function of Microsoft Excel.

## Abbreviations

AU: Arbitrary unit; FL: Fluorescence light; kb: Kilobase; MM: Minimal medium; N: Nitrogen; PCR: Polymerase chain reaction; TAG: Triacylglycerol; TLC: Thin layer chromatography; TAP: Tris-acetate-phosphate.

## Competing interests

The authors declare that they have no competing interests.

## Authors’ contributions

CC, BM, HMN, AB-A, SB, SC performed research. CC, BM, FB, GP, YL-B designed research. CC, BM, YL-B analyzed data. FB, GP commented on the manuscript. YL-B wrote the manuscript. All authors read and approved the final manuscript.

## Supplementary Material

Additional file 1 **Determination of oil content based on a densitometry method after separation on thin layer chromatography.** The standard used for TAG quantification was triheptadecanoin (C17:0 TAG). **(A)** Type I mutants under TAP condition. **(B)** Type II mutants under TAP-N condition. **(C)** Type III mutants under MM condition. Data are means of three biological replicates, error bars represent standard deviation. Click here for file
