# Supplementary material for: Development of a forward genetic screen to isolate oil mutants in the green microalga Chlamydomonas reinhardtii
Source: Biotechnol Biofuels. 2013 Dec 2;6:178. doi: 10.1186/1754-6834-6-178 (PMC4176504; doi:10.1186/1754-6834-6-178)

Additional file 1

Additional file 1: Determination of oil content based on a densitometry method after separation on Thin Layer Chromatography. The standard used for TAG quantification was triheptadecanoin (C17:0 TAG).

(**A**). Type I mutants under TAP condition.

(**B**). Type II mutants under TAP-N condition.

(**C**). Type III mutants under MM condition.

Data are means of three biological replicates, error bars represent standard deviation.

Abbreviations

AU, Arbitrary Unit; FL, Fluorescence Light; MM, Minimal Medium; N, Nitrogen; TAG, TriAcylGlycerol; TLC, Thin Layer Chromatography; TAP, Tris-Acetate-Phosphate.

Additional file 1

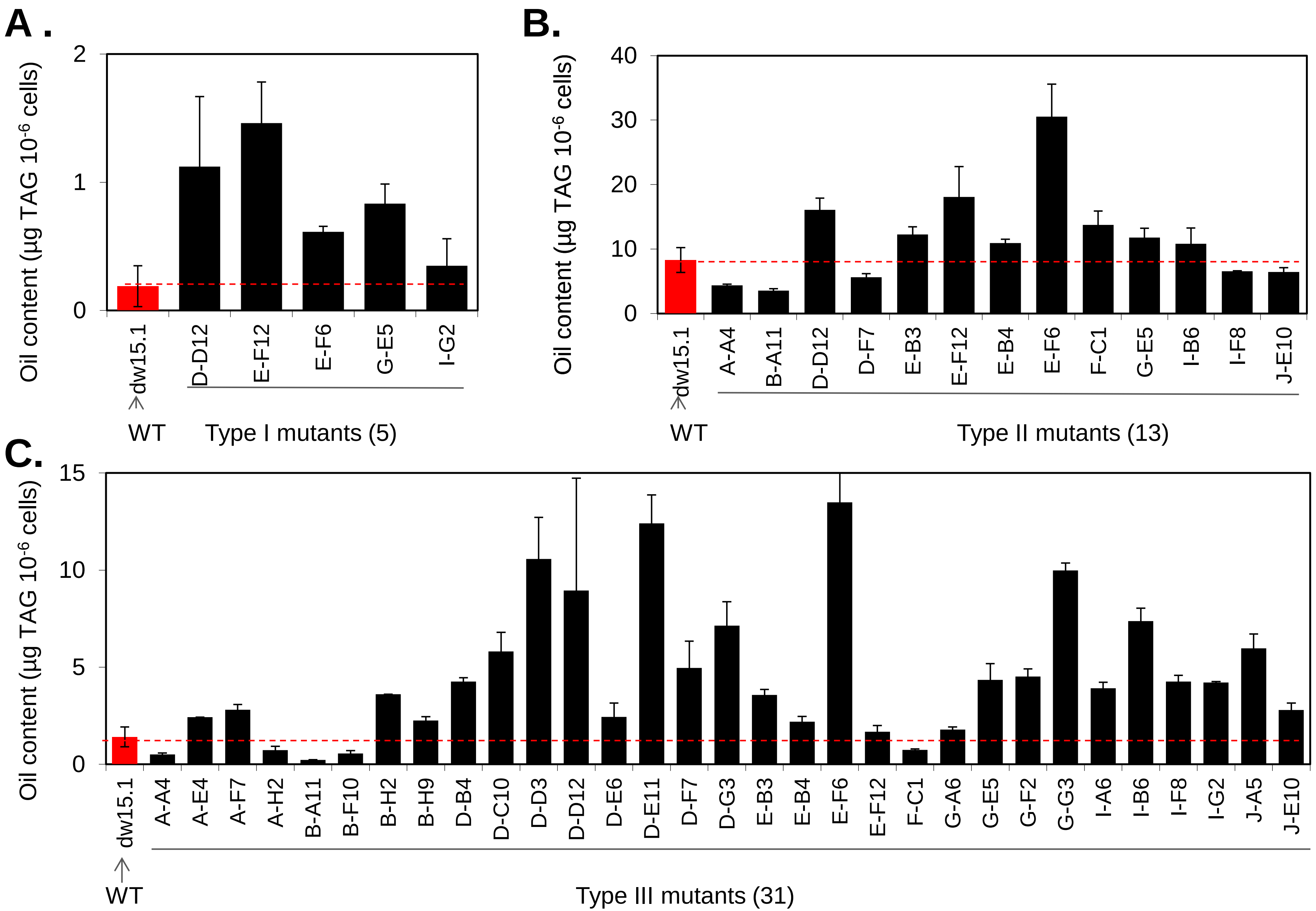

Supplement: Additional file 1 — Determination of oil content based on a densitometry method after separation on thin layer chromatography. The standard used for TAG quantification was triheptadecanoin (C17:0 TAG). (A) Type I mutants under TAP condition. (B) Type II mutants under TAP-N condition. (C) Type III mutants under MM condition. Data are means of three biological replicates, error bars represent standard deviation. [file 1754-6834-6-178-S1.pdf]
